# The Core–Shell Approach for Thermally Conductive and Electrically Insulating Polymer Nanocomposites: A Review

**DOI:** 10.1002/marc.202500078

**Published:** 2025-03-06

**Authors:** Antoine Bodin, Anne Coloigner, Thomas Pietri, Jean‐Pierre Simonato

**Affiliations:** ^1^ Université Grenoble Alpes CEA LITEN DTNM Grenoble F‐38000 France

**Keywords:** 3D printing, composites, heat dissipation, nanomaterials, thermal management

## Abstract

The development of new high‐performance materials in the field of polymer composites is becoming increasingly challenging as the requirements for real‐life applications evolve rapidly. In particular, the issue of heat dissipation in a multitude of devices has become a matter of critical importance due to the ever‐increasing compaction of electronic devices and the significant growth in power density stored in batteries. This calls for the development of novel solutions to enhance heat dissipation while preserving electrical insulation properties, particularly in light of safety concerns. In this context, polymer nanocomposites can play a significant role, as the incorporation of specific fillers can markedly improve their intrinsic properties, namely, low electrical conductivity, lightweightness, processability, and low cost. New fillers based on a core–shell structure have recently emerged. They are typically nanoscopic in size and synthesized through fine chemical processes to optimize their performance and ensure optimal cohesion with the polymer matrix. Nanocomposites based on core–shell nanofiller yield remarkable and highly promising outcomes, often exceeding the state of the art. This review article presents a comprehensive overview of these nanostructures and their applications, elucidating their significance and results, and discusses their role in achieving optimal heat dissipation.

## Graphical abstract

Core–shell nanoparticles open a promising route to thermally conductive and electrically insulating nanocomposites.

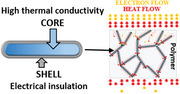

## 1 Introduction

The rapid growth of energy storage technologies and the evolution of new microelectronic systems have resulted in increasingly powerful systems. Such new systems simultaneously require greater miniaturization and further integration of components, thereby generating undesirable and potentially damaging heat for systems in operation. This has raised new challenges in further improving their performances, which have become critically dependent on the efficiency of thermal management.^[^ ^1^, ^2^ ^]^ A new class of heat dissipative structural materials needs to be developed. Besides the good thermal conductivity required for efficient heat dissipation, these materials must also demonstrate electrical insulation properties to avoid any interference with the electrical or electronic systems (short‐circuiting, over‐consumption of energy, signal propagation delay, etc.).^[^ ^1^, ^3^, ^4^ ^]^ Polymeric materials are particularly well suited for meeting these requirements, except for their low intrinsic thermal conductivity which is insufficient to dissipate heat efficiently. A promising strategy for improving thermal conductivity while preserving the electrical insulating properties of polymer materials is to incorporate thermally conductive yet electrically insulating filler.^[^ ^5^, ^6^, ^7^ ^]^

Ceramics appear as suitable fillers for this purpose, as they are intrinsically electrically insulating and thermally conductive.^[^ ^3^, ^8^ ^]^ The most commonly used ceramic fillers in thermally conductive polymer nanocomposites are either ceramic oxides such as silica (SiO_2_),^[^ ^9^ ^]^ alumina (Al_2_O_3_),^[^ ^10^ ^]^ or zinc oxide (ZnO),^[^ ^11^ ^]^ or non‐oxide ceramics such as aluminum nitride (AlN),^[^ ^12^ ^]^ silicon nitride (Si_3_N_4_),^[^ ^13^ ^]^ silicon carbide (SiC),^[^ ^14^ ^]^ or hexagonal boron nitride (BN)‐based nanostructures (hBN nanosheets, hBN nanotubes).^[^ ^15^, ^16^, ^17^, ^18^ ^]^ However, although ceramic materials exhibit intrinsic electrical insulation (absence of free electrons), they also tend to possess lower thermal conductivities than their carbon or metallic counterparts.^[^ ^19^, ^20^, ^21^, ^22^ ^]^ Carbon‐based and metallic fillers generally exhibit very high thermal conductivity, but they also present a too high electrical conductivity. They are mainly used for applications that do not require advanced electrical insulation properties. Carbon‐based fillers include graphite,^[^ ^23^ ^]^ graphene,^[^ ^24^ ^]^ graphene oxide (GO),^[^ ^25^ ^]^ carbon fibers (CF),^[^ ^26^ ^]^ and multi‐walled carbon nanotubes (MWCNT).^[^ ^22^, ^27^ ^]^ Among metallic nanoparticles, the most widely used are made of gold (Au),^[^ ^28^ ^]^ copper (Cu),^[^ ^29^ ^]^ and silver (Ag),^[^ ^30^ ^]^ because they exhibit the highest thermal conductivities among metals. To take advantage of this high thermal conductivity while achieving a high level of electrical insulation, it has then become necessary to develop a new type of fillers: core–shell (nano)materials.

**Figure 1:**
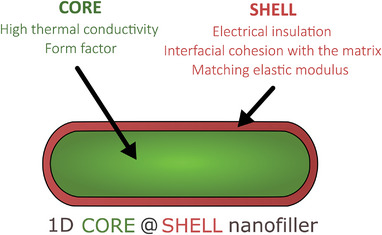
Scheme of a core–shell 1D nanostructure for the fabrication of thermally conductive and electrically insulating nanocomposites.

Core–shell technology consists in the coating of one particular compound (“core”) by another of a different chemical nature (“shell”). This type of heterostructure has been designed to electrically insulate highly thermally conductive cores that also exhibit undesired electrical conduction properties (e.g., carbonaceous or metallic particles), as shown in **Figure** **1**.

This strategy is also used concomitantly as an alternative functionalization method to improve dispersion and chemical compatibility between a filler and the polymer matrix. To limit electrical conductivity of carbon‐based or metallic core materials, the most commonly used shells are made of ceramic materials such as SiO_2_,^[^ ^31^, ^32^ ^]^ Al_2_O_3_,^[33,^ ^34^ ^]^ or hBN.^[^ ^35^, ^36^ ^]^ In comparison with the hybrid filler strategy where different shapes, sizes, and natures are mixed together,^[^ ^37^, ^38^, ^39^, ^40^ ^]^ core–shell technology enables the synergy effect to be focused within a single filler, minimizing the filler load required to achieve a targeted thermal conductivity. When possible, nanofillers with high aspect ratios such as 1D nanostructures (nanofibers, nanotubes, nanowires, nanorods, etc.) are preferably used as they ensure a thermal conductivity enhancement at lower loading ratios through a 3D percolative network, thereby avoiding high viscosities and processability issues.^[^ ^30^, ^41^, ^42^ ^]^ In the literature, authors generally adopt the “core@shell” notation to define core–shell structures, “core” and “shell” indicating the chemical natures of the two elements. After a brief reminder of the various general concepts dealing with thermal conductivity in polymer nanocomposites, which have already been extensively reviewed over the past few years,^[^ ^3^, ^5^, ^6^, ^19^, ^20^, ^21^, ^37^, ^41^, ^43^ ^]^ this review focuses specifically on polymer nanocomposites filled with thermally conductive and electrically insulating core@shell fillers.

**Figure 2:**
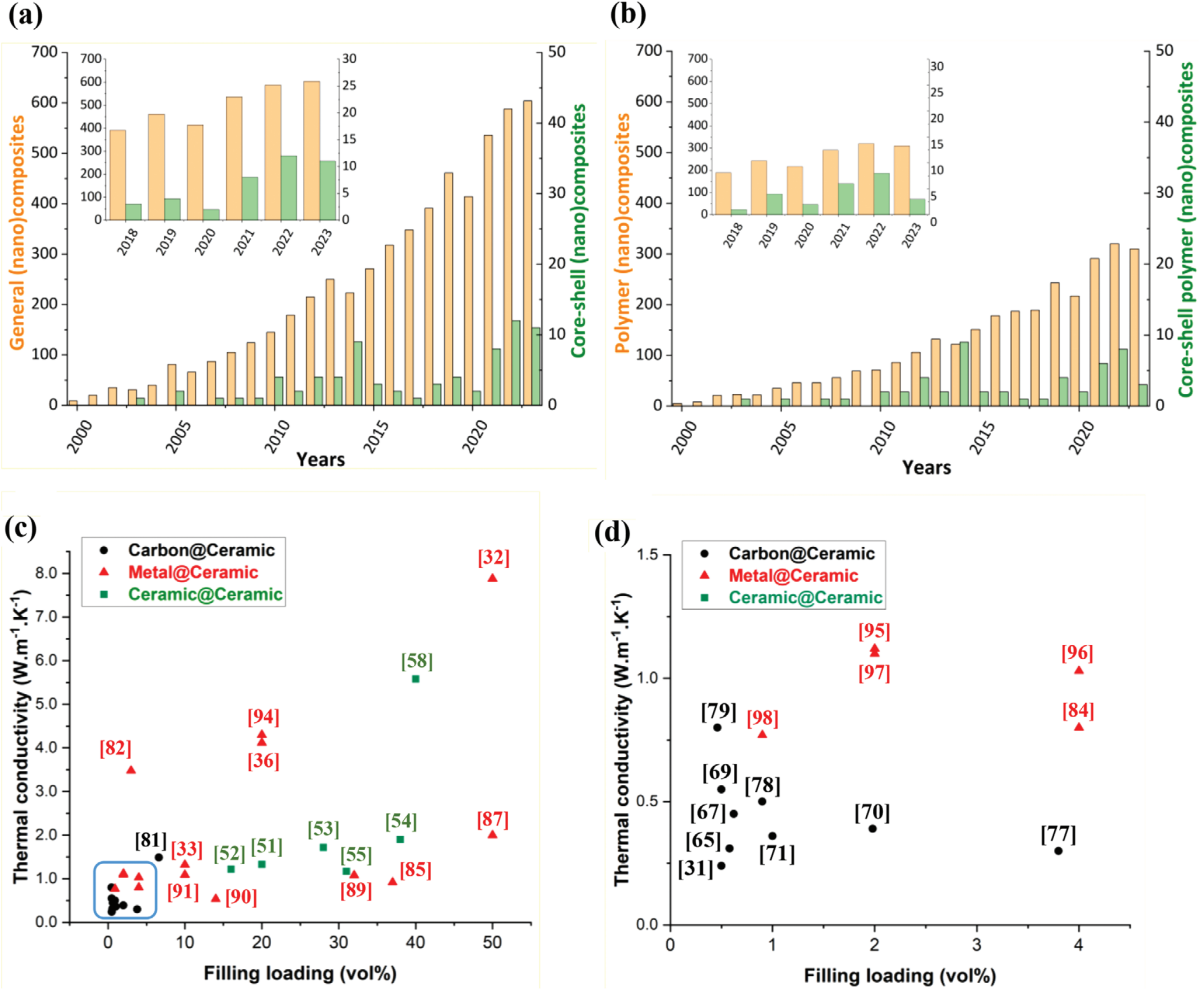
Evolution of the number of publications regarding a) thermally conductive and electrically insulating nanocomposites and b) by considering polymer matrix as an element of the investigation (Scopus). c,d) Graphics representing the state of the art for different core–shell systems.

A bibliometric survey, presented in **Figure** **2**a,b, was performed to report the significant recent and growing interest in nanocomposites core–shell systems with thermal conductivity and electrical insulation properties. Ceramic@ceramic, carbon@ceramic, metal@ceramic, and core@polymer fillers are reviewed and discussed, and their use in several thermal management applications is exemplified. The thermal conductivity of some core–shell nanocomposites is presented in Figure 2c,d.

## 2 Thermal Conductivity in Polymer Nanocomposites

Thermal conduction results from the transfer of thermal energy within an object of varying temperatures. It can be described, in a homogeneous and isotropic medium, by the Fourier's law [Equation (1)], where $\vec{\varphi }$ represents the heat flux density, λ the thermal conductivity, and $\vec{grad}\;T$ the temperature gradient oriented in the direction of temperature increase.

(1) $$\vec{\varphi }=-\lambda .\vec{grad}T$$

Thermal conductivity (λ) represents the intrinsic ability of a material to transfer heat. It describes the rate at which heat propagates through a material when subjected to a temperature change. Thermal conductivity values mainly depend on the material's chemical composition, density, and crystalline structure. They can be calculated using Equation (2), where α, ρ, and *C*_p_ describe, respectively, the thermal diffusivity, density, and specific heat capacity of the material. These data are commonly obtained experimentally. Thermally conductive materials possess high thermal conductivity and transfer heat rapidly, while insulating materials block heat transfer and demonstrate low thermal conductivity.

(2) $$\lambda =\alpha .\rho .C_{p}$$

Heat conduction in solids takes place via charge carriers (electrons, holes) or phonons (quanta of atomic lattice vibration energy). In electrically conductive solids, free electrons are the main contributors to heat transfer. On the other hand, in electrically insulating solids, devoid of free electrons (ceramics or polymers), heat conduction is mainly ensured by the contribution of phonons, generated by the vibration of the crystalline lattice in which atoms and molecules vibrate around their equilibrium position.^[^ ^19^ ^]^ In a nonmetallic material, the more efficient the phonon transport, the higher its thermal conductivity. The thermal conductivity is expressed in Equation (3) as the product of the phonon group velocity *v*, the mean free path *l* and C_v_ which represents the material's volumetric heat capacity.^[^ ^20^ ^]^

(3) $$\lambda =\frac{C_{v}.v.l}{3}$$

**Figure 3:**
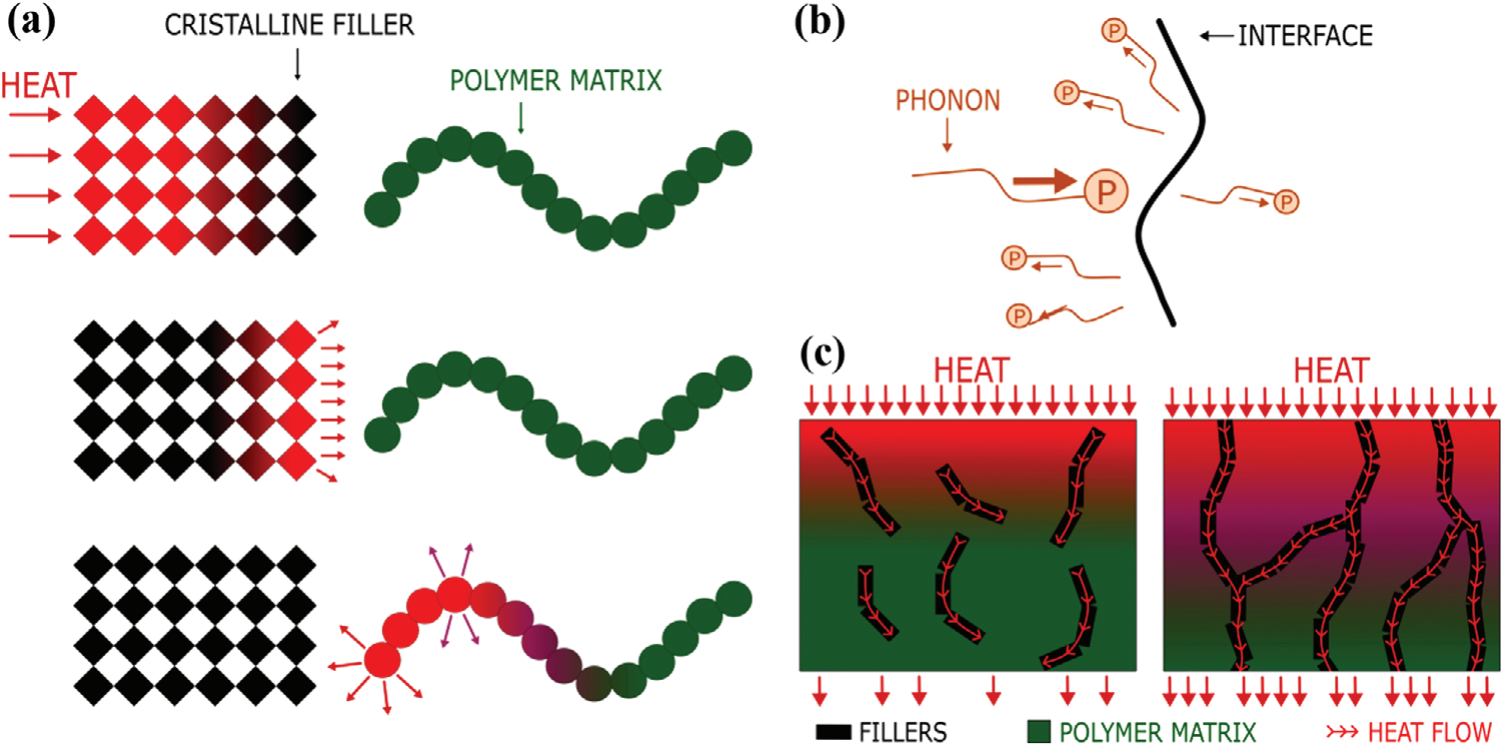
Schematic diagrams of a) the discontinuity of heat transfer at the filler–matrix interface leading to phonon scattering, b) the change of energy and direction of an incident phonon at an interface causing phonon‐boundary scattering, and c) the effect of dispersion of the fillers on the formation of an efficient thermally conductive network.

If phonon transport is disrupted for whatever reason, then thermal conductivity is drastically reduced. This disturbance is commonly referred to as phonon scattering and occurs whenever a phonon undergoes a change of direction, momentum or energy (**Figure** **3**b). In a purely crystalline filler, phonons move relatively efficiently, as organized zones of atoms favor their propagation. Thermal energy is, therefore, optimally transferred from one end of the material to the other, if the crystal is considered perfect.^[^ ^41^ ^]^ However, at the interface with the polymer matrix, an acoustic mismatch between the crystalline filler and the amorphous polymer inevitably leads to phonon scattering (Figure 3a). This phenomenon is responsible for the appearance of interfacial thermal resistances, which are behind the overall reduction in thermal conductivity. Thermal boundary resistance (also known as Kapitza resistance^[^ ^44^ ^]^) is the consequence of phonon scattering resulting from the change in atomic structure between the two phases. Thermal contact resistance is associated with incomplete or poor‐quality filler–matrix adhesion, creating porosities that are detrimental to the proper circulation of phonons.

The thermal conductivity of polymer nanocomposites is also governed by the level of dispersion of the fillers throughout the polymer matrix. Poor dispersion is often caused by filler agglomeration or segregation. Particle clusters are formed because of Van der Waals interaction forces and the surface energy of nanofillers. These particle clusters increase contact resistances and reduce the chances of forming conductive paths for phonons (Figure 3c), two main factors responsible for limited thermal conductivity.^[^ ^45^, ^46^ ^]^

The morphology of the nanoparticles plays a crucial role in the thermal conductivity of nanocomposites. The formation of a 3D thermally conductive network occurs around a certain loading rate, which varies according to the size and form factor of the fillers. In addition to their intrinsic properties and the importance of interfacial interactions with the matrix to avoid phonon scattering, the fillers have to reach percolation at a low critical loading rate to form an effective thermally conductive network. For nanofillers of the same chemical composition and at equivalent loading rates, 0D particles appear to be much less effective than their 1D or 2D counterparts. This is due to a larger specific surface area with the matrix compared to their 1D or 2D counterparts of equivalent volume, and the fact that percolation can only be achieved at high concentration in terms of required contact points between 0D particles. Conversely, a high form factor (1D, 2D fillers) enables the creation of long‐range, more efficient conductive pathways with far fewer filler–matrix interfaces, allowing the formation of high performance thermally 3D percolative networks at relatively low loading rates. This also avoids high viscosities and processing issues, as well as altering the mechanical properties of the polymer composite.

A growing number of applications (electronics, LEDs, batteries, etc.) require not only good heat dissipation, but also sufficient electrical insulation to avoid possible electrical disturbances. Electrically insulating materials do not display free flowing electrons, and exhibit an electrical resistivity of at least 10^9^ Ω cm (such as polymers or ceramics). On the contrary, electrically conductive materials include metals and carbon‐based materials, where electrons can flow freely. Due to their intrinsic electrically insulating properties, ceramic fillers have been used to develop thermally conductive and electrically insulating polymer nanocomposites. However, the thermal conductivity of ceramic materials is generally inferior to that of metallic or advanced carbon‐based materials. To make an electrically conductive composite insulating, it is thus necessary to find a way of blocking the flow of electrons while maintaining a good overall thermal conductivity. The dielectric properties of such materials are widely illustrated in the literature.^[^ ^47^, ^48^, ^49^ ^]^ This is why thermally conductive and electrically insulating core–shell fillers with a metallic or carbon core and a ceramic or polymer shell are currently the focus of major development efforts.

## 3 Core@Shell Fillers in Thermally Conductive and Electrically Insulating Polymer Nanocomposites

### 3.1 Ceramic@Ceramic Fillers

**Figure 4:**
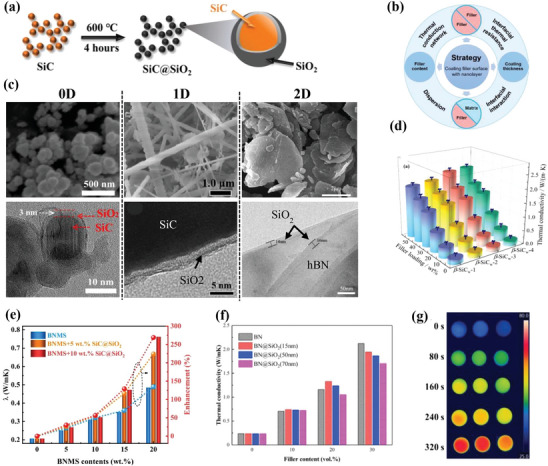
a) Schematic representation of the oxidation process of SiC (left) and SiC@SiO_2_ powders (right).^[^ ^50^ ^]^ b) Effects and mechanism of the nanolayer coating strategy on the thermal conductivity of composites.^[^ ^51^ ^]^ c) Microscopic images of ceramic fillers coated with a ceramic shell according to different structural dimensions: (0D) spherical silicon carbide particles coated with silica (SiC@SiO_2_),^[^ ^50^ ^]^ (1D) silicon carbide whiskers coated with silica (β‐SiC_w_@SiO_2_),^[^ ^52^ ^]^ (2D) hexagonal boron nitride coated with silica (hBN@SiO_2_).^[^ ^51^ ^]^ Filler content dependence of thermal conductivity for different ceramic@ceramic systems: d) SiC@SiO within a PVDF matrix,^[^ ^53^ ^]^ e) BNMS/SiC@SiO_2_within an epoxy matrix,^[^ ^50^ ^]^ and f) hBN@SiO_2_ within an epoxy matrix.^[^ ^51^ ^]^ g) Infrared thermal images of epoxy‐BNMS/SiC@SiO_2_ at 20 wt% of BNMS, with 10, 5, and 0 wt% of SiC@SiO_2_ from the left to the right the respectively.^[^ ^50^ ^]^ (a,c,e,g) Reproduced with permission.^[^ ^50^ ^]^ Copyright 2022, Elsevier. (b,c,f) Reproduced with permission.^[^ ^51^ ^]^ Copyright 2021, Elsevier.

In the particular case of ceramic@ceramic nanostructures where the core is already electrically insulating by itself, the shell is engineered only to provide surface functionalization to the core material in order to increase chemical compatibility with the polymer matrix and homogeneous dispersion, thereby decreasing interfacial thermal resistance and improving thermal conductive networks. Some examples of ceramic@ceramic core–shell nanostructures are presented in **Figure** **4**.

#### 3.1.1 0D Fillers

The most common 0D ceramic cores are either made of silicon carbide (SiC), silica (SiO_2_), or alumina (Al_2_O_3_). Hwang et al.^[^ ^54^ ^]^ synthesized SiC@Al_2_O_3_ core–shell particles by firstly activating the SiC surface via HF etching and H_2_O_2_ treatment. Then, the Al_2_O_3_ layer was deposited using aluminum isopropoxide (AlIP) in solution followed by a heat treatment. The SiC@Al_2_O_3_ fillers were then introduced inside an epoxy matrix to form epoxy‐SiC@Al_2_O_3_ polymer composites. It was shown that the epoxy composite using SiC@Al_2_O_3_ fillers exhibited higher thermal conductivity than the reference composite using raw SiC particles. At 65 wt% loading (equivalent to 38.4 vol% in this particular case), epoxy‐SiC@Al_2_O_3_ composites led to a thermal conductivity of 1.9 W m^−1^ K^−1^ while epoxy‐SiC composites exhibited a lower value of 1.5 W m^−1^ K^−1^. The higher thermal conductivity observed for epoxy‐SiC@Al_2_O_3_ composites was explained by the improvement of the filler–matrix interfacial adhesion leading to a decrease in thermal resistance. Moreover, the Al_2_O_3_ layer seemed to improve the electrical insulation of the composites since the electrical conductivity measured on epoxy‐SiC@Al_2_O_3_ samples was lowered compared to epoxy‐SiC composites. Zhao et al.^[^ ^50^ ^]^ also used SiC particles as core material for SiC@SiO_2_ core–shell nanofillers. The SiO_2_ shell was synthesized by heat‐treating SiC nanoparticles for 4 h at 600 °C under air atmosphere. In this particular case, the SiC@SiO_2_ 0D ceramic core–shell nanoparticles were mixed together with boron nitride microsphere (BNMS) as a co‐filler in an epoxy matrix. With the addition of 10 wt% of SiC@SiO_2_ core–shell nanoparticles to the epoxy‐BNMS (20 wt%) composite, the thermal conductivity increased up to 0.76 W m^−1^ K^−1^. This improvement in thermal conductivity was explained by a synergetic effect between the BNMS and the SiC@SiO_2_ nanoparticles, where more heat transfer paths and a well‐constructed thermal percolation transport network were achieved. Rybak et al.^[^ ^55^ ^]^ reported two kinds of ceramic@ceramic core–shell particles loaded inside an epoxy matrix: SiO_2_@Si_3_N_4_ and Al_2_O_3_@AlN. These core–shell fillers were synthesized by a carbothermal reduction and nitridation process (CTRN). In both cases, the thermal conductivity value was increased compared to the one measured on epoxy‐SiO_2_ and epoxy‐Al_2_O_3_ composites, respectively, and reached 1.17 W m^−1^ K^−1^ for the epoxy‐Al_2_O_3_@AlN composite loaded at 31 vol%. This thermal conductivity enhancement was also explained by the reduction of the interfacial thermal resistance through filler‐matrix interface improvement, especially because Si_3_N_4_ and AlN exhibit higher intrinsic thermal conductivities (100–200 and 100–320 W m^−1^ K^−1^, respectively) compared to their respective core materials (1.5 W m^−1^ K^−1^ for SiO_2_ and 20–35 W m^−1^ K^−1^ for Al_2_O_3_).

#### 3.1.2 1D Fillers

Ceramic@ceramic core–shell fillers are also available as 1D fillers. Zhou et al.^[^ ^53^ ^]^ and Cao et al.^[^ ^52^, ^56^ ^]^ both studied SiC@SiO_2_ nanowires as fillers in a PVDF matrix. In both studies, the authors found that the thermal conductivity was improved using SiC@SiO_2_ core–shell nanofillers instead of raw SiC nanowires. The highest reported values were 1.72 W m^−1^ K^−1^ at 40 wt% (27.7 vol%) loading for the study of Zhou et al. and 1.22 W m^−1^ K^−1^ at 24.4 wt% (15.6 vol%) loading for the study of Cao et al. The thermal conductivity enhancement was ascribed to the improvement of filler–polymer interfacial interactions through hydrogen bonding between PVDF and the Si─OH groups of the SiO_2_ shell. The significantly improved interfacial compatibility greatly reduces defects and voids within the boundary zone. This, in turn, mitigates the phonon scattering phenomenon, thereby facilitating phonon transport across the interface. Moreover, the SiO_2_ shell provided a better electrical insulation and improved dielectric properties to the PVDF‐SiC@SiO_2_ composite compared to the PVDF‐SiC composite thanks to its remarkable insulating behavior.

#### 3.1.3 2D Fillers

Most ceramic fillers are based on 2D hexagonal boron nitride (hBN) because it exhibits the highest intrinsic thermal conductivity among ceramics (200–300 W m^−1^ K^−1^). However, they often suffer from poor interfacial interactions with the polymer matrix, resulting in low thermal conductivity values for the composite. Yan et al.^[^ ^57^ ^]^ grafted Al_2_O_3_ onto the surface of boron nitride nanosheets (BNNS) to form BNNS@Al_2_O_3_ thermally conductive and electrically insulating 2D core–shell nanofillers. Incorporated inside a silicone rubber matrix, this hybrid core–shell nanofiller provided a thermal conductivity of 2.86 W m^−1^ K^−1^ at 30 wt% loading in the in‐plane direction, while maintaining a good electrical resistivity of 5.80 × 10^11^ Ω cm. Tang et al.^[^ ^58^ ^]^ coated hBN particles with silica through a sol‐gel process after activation of the hBN surface via physical adsorption of polyvinylpyrrolidone (PVP). The resulting hBN@SiO_2_ core–shell particles with a silica layer thickness of ≈20–30 nm were introduced in a PMMA matrix to form PMMA‐hBN@SiO_2_ composites via solution mixing. At 40 vol% loading, PMMA‐hBN@SiO_2_ composites exhibited a thermal conductivity of 5.58 W m^−1^ K^−1^, which was higher than the PMMA‐hBN reference composite. The better filler–matrix interfacial interaction provided by the SiO_2_ interlayer allowed reducing the interfacial thermal resistance, thus favoring phonon transport at the interface, and increasing the overall thermal conductivity. This superior filler–matrix cohesion also contributed to strengthen the composite with an improvement in the ultimate tensile strength (almost doubled) compared to the PMMA‐hBN composite. Similarly, Liang et al.^[^ ^51^ ^]^ synthesized SiO_2_ nanolayers on hBN using a sol‐gel method with varying initial concentrations of TEOS (SiO_2_ precursor) to obtain different SiO_2_ thicknesses varying from 15 to 70 nm. Once purified, the resulting hBN@SiO_2_ core–shell fillers were introduced in an epoxy matrix to obtain epoxy‐hBN@SiO_2_ composites. At 20 vol% filler loading, the epoxy composites filled with the hBN@SiO_2_ particles with a SiO_2_ thickness of 15 nm exhibited a higher thermal conductivity than that of the composites filled with raw hBN (1.33 vs 1.16 W m^−1^ K^−1^, respectively). However, at higher SiO_2_ thicknesses (50 and 70 nm), the thermal conductivity enhancement inverted and the thermal conductivity value decreased down to 1.24 and 1.06 W m^−1^ K^−1^, respectively. For SiO_2_ thicknesses below 15 nm, the thermal conductivity enhancement can mainly be ascribed to the improvement of the wettability between the filler and the polymer matrix thanks to hydroxyl groups present at the surface of hBN@SiO_2_ fillers. This leads to a better interfacial interaction, reducing phonon scattering at the interface. On the other hand, for samples with thicker SiO_2_ shells (50 and 70 nm), the low intrinsic thermal conductivity of SiO_2_ (≈1.5 W m^−1^ K^−1^) gradually restrains the heat transfer at the filler–matrix interface by reducing the group velocity of phonons, thus leading to lower thermal conductivities. Awais et al.^[^ ^59^ ^]^ studied the synergetic effects between surface‐modified hBN and TiO_2_@SiO_2_ fillers within an epoxy matrix on the overall thermal conductivity and electrical insulation of the prepared nanocomposite. The TiO_2_@SiO_2_ core–shell nanostructures were synthesized by a sol‐gel reaction in the presence of TEOS on the surface of TiO_2_ particles, whereas the surface of hBN was modified with dopamine‐HCl. Both fillers were incorporated in various proportions in an epoxy matrix. The authors found that the thermal conductivity was improved by increasing the proportion of hBN, whereas the electrical insulation properties were enhanced in the presence of TiO_2_@SiO_2_ core–shell nanostructures. At 10 wt% of hBN and 3 wt% of TiO_2_@SiO_2_, the thermal conductivity of the nanocomposite reached 0.67 W K^−1^ m^−1^. Some results concerning ceramic@ceramic core–shell filled polymer nanocomposites are gathered in **Table** **1**.

**Table 1:** Thermal conductivities and electrical resistivities of polymer nanocomposites made with ceramic@ceramic core–shell nanofillers.

Polymer matrix	Core–shell filler	Surface activation of the core	Nanocomposite processing route	Loading ratio [wt%‐vol%]	Thermal conductivity of the pure polymer matrix [W m^−1^ K^−1^]	Thermal conductivity of the nanocomposite [W m^−1^ K^−1^]	Electrical resistivity [Ω cm]	Ref.
Epoxy	SiC@Al_2_O_3_	HF etching + H_2_O_2_ treatment	Blending	65‐38	*n.a*.	1.9	*n.a*.	[54]
Epoxy	Al_2_O_3_@AlN	–		*n.a*.‐31		1.17	*n.a*.	[55]

PVDF	SiC@SiO_2_	–	Solvent	40‐28	0.20	1.72	*n.a*.	[53]
				24‐16		1.22	*n.a*.	[52]

SR	BNNS@Al_2_O_3_	–	Blending	30‐*n.a*.	1.01	2.86	5.80 × 10^11^	[57]

PMMA	hBN@SiO_2_	PVP (amphiphile polymer)	Solvent	*n.a*.‐40	0.16	5.58	*n.a*.	[58]

Epoxy	hBN@SiO_2_	–	Solvent	*n.a*.‐20	0.25	1.33	*n.a*.	[51]

### 3.2 Carbon@Ceramic Fillers

**Figure 5:**
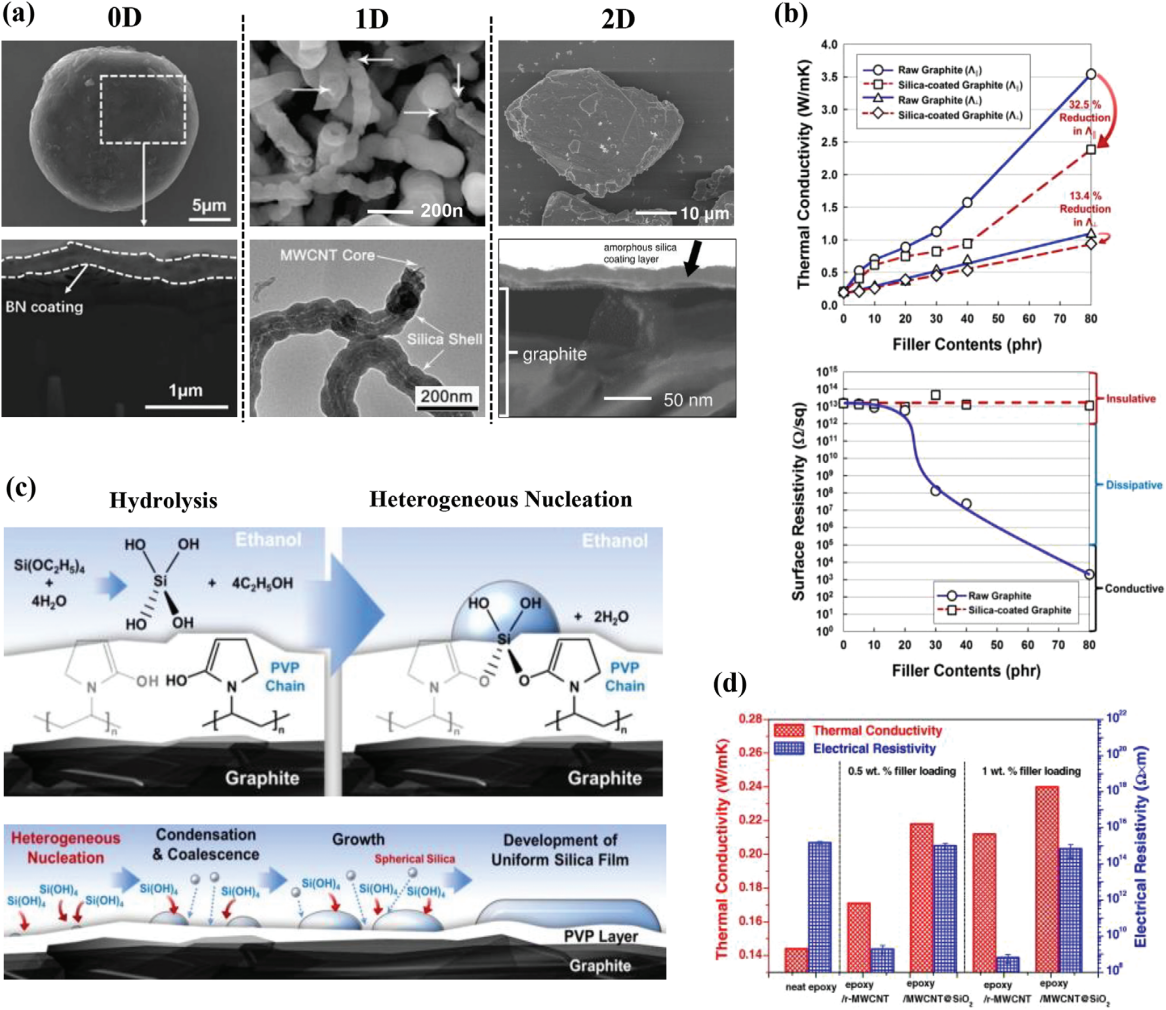
Carbon fillers with a ceramic coating can improve electrical insulation and maintain a high thermal conductivity in nanocomposites. a) Microscopic images of carbon fillers coated with a ceramic shell according to different structural dimensions: (0D) spherical carbon black particles coated with boron nitride (SR@BN),^[^ ^35^ ^]^ (1D) multi‐walled carbon nanotubes coated with silica (MWCNT@SiO_2_),^[^ ^31^ ^]^ (2D) graphite nanosheets coated with silica (Graphite@SiO_2_).^[^ ^60^ ^]^ Filler content dependence of thermal conductivity and surface resistivity for different carbon@ceramic systems: b) Graphite@SiO_2_ within a polyester elastomer matrix^[^ ^61^ ^]^ and d) MWCNT@SiO_2_ within and an epoxy matrix.^[^ ^31^ ^]^ c) Surface modification scheme of graphite via PVP functionalization and TEOS hydrolysis to produce a silica layer. (a) Reproduced with permission.^[^ ^35^ ^]^ Copyright 2023, Elsevier. (a,d) Reproduced with permission.^[^ ^31^ ^]^ Copyright 2021, Elsevier. (a) Reproduced with permission.^[^ ^60^ ^]^ Copyright 2014, Elsevier. (b,c) Reproduced with permission.^[^ ^61^ ^]^ Copyright 2013, Elsevier.

Of the three types of filler available to improve the thermal conductivity of polymer nanocomposites, carbon‐based fillers exhibit the highest theoretical intrinsic thermal conductivities. Unfortunately, they are also very good electrical conductors. Several strategies for producing electrically insulating coatings on the surface of carbonaceous fillers have therefore been developed to overcome this issue. Some examples of carbon@ceramic core–shell nanostructures are presented in **Figure** **5**.

#### 3.2.1 0D Fillers

0D carbon‐based particles such as carbon black are usually not used as core material for core–shell fillers because they do not exhibit very high thermal conductivity compared to their 1D or 2D counterparts. However, some authors still consider coating 0D carbon particles with ceramic shells. Kong et al.^[^ ^35^ ^]^ reported a boron nitride coating on the surface of spherical graphite (SG) particles. The SG surface was firstly activated by grafting polydopamine (PDA) before coating BN layers using boric acid and melamine in solution, followed by two thermal annealing processes (under ammonia then dry nitrogen atmosphere). The resulting SG@BN core–shell particles were finally incorporated into a silicon rubber (SR) matrix to form SR‐SG@BN composites. Thermal conductivity of such composite reached a value of 3.65 W m^−1^ K^−1^ at a high 70 vol% loading. The BN conformal coating on SG@BN core–shell fillers proved efficient to improve electrical properties such as breakdown voltage, electrical resistivity, and dielectric properties.

#### 3.2.2 1D Fillers

Most 1D carbon‐based cores are either made of MWCNT or CF. The majority of ceramic shells regarding carbon@ceramic core–shell fillers with MWCNT cores is made of silica SiO_2_.^[^ ^31^, ^62^, ^63^, ^64^, ^65^ ^]^ In these studies, the SiO_2_ shell was synthesized through a sol‐gel process using TEOS as a precursor after activating the MWCNT surface beforehand.

The easiest and most common method to activate MWCNTs is through oxidation processes, which create hydroxyl grafting sites on their surface. This preliminary step is usually performed under strong acidic conditions using a combination of hot concentrated nitric acid (HNO_3_) and sulfuric acid (H_2_SO_4_), with a volume ratio *V*_HNO3_/*V*_H2SO4_ from 3:1^[^ ^31^, ^65^ ^]^ to 1:3.^[^ ^62^, ^64^ ^]^ Thanks to the hydroxyl groups formed at the surface of MWCNTs following this severe acidic treatment, it is possible to make hydrolyzed TEOS molecules react at the surface of MWCNT‐OH through condensation reactions performed under basic conditions (usually using NH_4_OH as a catalyst). Then, these MWCNT@SiO_2_ core–shell nanofillers are incorporated in a polymer matrix to form polymer‐MWCNT@SiO_2_ nanocomposites. Cui et al.^[^ ^31^ ^]^ studied epoxy‐MWCNT@SiO_2_ nanocomposites and reported a thermal conductivity value of 0.24 W m^−1^ K^−1^ with an electrical resistivity value of 6.90 × 10^16^ Ω cm at 1 wt% (0.5 vol%) loading ratio. Zhao et al.^[^ ^65^ ^]^ used polyurethane (PU) as the polymer matrix and reported a thermal conductivity value of 0.31 W m^−1^ K^−1^ for an electrical resistivity of 1.00 × 10^14^ Ω cm at 1 wt% (0.58 vol%) loading ratio. Hu et al.^[^ ^62^ ^]^ chose to mix 1D MWCNT@SiO_2_ core–shell fillers with modified hBN 2D ceramic filler (1:4 mass ratio), creating a hybrid thermally conductive network within a PVDF matrix. The bridging between hBN 2D fillers offered by the presence of 1D MWCNT@SiO_2_ fillers led to enhanced thermal conductivity with values reaching 1.51 W m^−1^ K^−1^ at 25 wt% (≈22 vol%) loading ratio, while exhibiting satisfying electrical resistivity of 3.50 × 10^12^ Ω cm. Based on the same approach, Wu et al.^[^ ^66^ ^]^ prepared MWCNT@BN/SiO_2_ core–shell nanostructures within a PVDF matrix to observe the synergetic effects of 1D MWCNT fillers and 2D BN dimensions. At 30 wt% hBN loading, the PVDF‐MWCNT@BN/SiO_2_ composite exhibits an electrically insulating behavior with an electrical resistivity of 3.6 × 10^15^ Ω cm. Out‐of‐plane and in‐plane thermal conductivities were measured at 0.508 and 1.564 W m^−1^ K^−1^, respectively. Further functionalization of the SiO_2_ shell is also possible by making smart use of the pendant silanol groups. Yu et al.^[^ ^67^ ^]^ used aminopropyltriethoxysilane (APTES) and bismaleimide (BMI) to form a double shell at the surface of MWCNTs. The organic compound of BMI is believed to enhance the compatibility of MWCNT with the epoxy matrix, leading to better thermal conductivity values up to 0.45 W m^−1^ K^−1^ at 1.25 wt% (≈0.62 vol%) loading ratio. Thanks to this engineered double shell, not only was the thermal conductivity improved, but the electrical resistivity was also preserved (2.90 × 10^15 ^Ω cm).

However, the formation of hydroxyl groups using such severe surface activation processes often comes at the expense of the structural integrity of the MWCNT crystalline structure. Several defects are thus created at the surface of MWCNTs, thereby increasing phonon scattering within the nanotube, which in turn results in reducing drastically their thermal conductivity.^[^ ^41^, ^68^ ^]^ For this reason, some authors proposed to use an alternative approach based on a noncovalent activation process. This softer activation process relies on the use of physical interactions between the MWCNT surface and the intermediate substance. Wang et al.^[^ ^69^ ^]^ used the CTAB surfactant (cetyltrimethylammonium bromide) to functionalize the surface of MWCNTs. The silanization reaction then proceeds in the same way as for oxidatively activated MWCNTs (using TEOS in basic conditions). In an epoxy matrix, they obtained a thermal conductivity value of 0.55 W m^−1^ K^−1^ at 1 wt% (≈0.5 vol%) loading ratio, which is more than twice better than the value obtained by Cui et al.^[^ ^31^ ^]^ who used a severe activation process, while preserving the electrical insulation property of the epoxy‐MWCNT@SiO_2_ nanocomposite with an electrical resistivity of 2.00 × 10^12^ Ω cm.

SiO_2_ shells aside, some studies focused on other ceramic shells for MWCNT@ceramic core–shell nanofillers. After an oxidative treatment performed with concentrated nitric acid to primarily functionalize the surface of MWCNTs with ─OH and ─COOH groups, Yan et al.^[^ ^70^ ^]^ managed to prepare MWCNT@BN core–shell nanostructures through the use of boric acid H_3_BO_3_ and urea in ethanol. The BN shell was then obtained after a thermal treatment in a furnace (3 h at 900 °C under NH_3_ atmosphere). Polyimide (PI)‐MWCNT@BN nanocomposites were then synthesized through in situ polymerization of diamine and dianhydride monomers followed by thermal imidization. At 3 wt% (≈1.98 vol%) MWCNT@BN loading, the thermal conductivity of the nanocomposite reached a value of 0.39 W m^−1^ K^−1^, which was five times higher than PI‐MWCNT films. This thermal conductivity enhancement was explained by the improvement of the dispersion, and the improved interface between the MWCNT@BN nanofiller and the PI matrix. The BN shell allowed the electrical resistivity of the PI‐MWCNT@BN nanocomposite to be higher than the reference PI‐MWCNT nanocomposite (up to 7.69 × 10^9^ Ω cm). Du et al.^[^ ^71^ ^]^ synthesized MWCNT@MgO core–shell nanostructures after prior MWCNT surface activation by acid treatment. For the MgO shell to be fabricated at the surface of the oxidized MWCNT, a MgCl_2_ solution was added to the nanotube suspension prior to addition of ammonia to convert Mg^2+^ ions into Mg(OH)_2_. A thermal treatment for 4 h at 600 °C under argon atmosphere eventually yielded MWCNT@MgO core–shell nanostructures with an ≈15‐nm MgO shell thickness. These nanofillers were added in an epoxy resin, then thermally cured to finally obtain epoxy‐MWCNT@MgO nanocomposites. The MgO layer promoted the interfacial interaction between MWCNTs and the epoxy matrix, leading to an improvement of the thermal conductivity up to 0.36 W m^−1^ K^−1^ at 2 wt% (≈1 vol%) loading. The electrical insulation properties of the epoxy matrix were preserved in the epoxy‐MWCNT@MgO nanocomposite thanks to the insulating MgO ceramic shell, the electrical resistivity being measured at 2.40 × 10^16^ Ω cm.

CFs are seldom used as cores for 1D carbon@ceramic core–shell nanostructures. Zhang et al.^[^ ^72^ ^]^ deposited a SiC shell on MWCNT via a chemical vapor deposition (CVD) method to fabricate CF@SiC core–shell nanofillers. Following their fabrication, these nanofillers were introduced in a PDMS matrix to form PDMS‐CF@SiC composites. At 20 wt% loading, the thermal conductivity reached 0.98 W m^−1^ K^−1^ while the electrical resistivity was at the edge of the insulating zone (3.91 × 10^8^ Ω cm). Huang et al.^[^ ^73^ ^]^ synthesized CF@BN core–shell nanostructures by a cooling precipitation method and a thermal treatment. After incorporation in a silicon rubber by an extrusion process, CF@BN nanofillers provided to the nanocomposite a thermal conductivity value of 12.06 W m^−1^ K^−1^ and an electrical resistivity of 5 × 10^9^ Ω cm at 20 wt% loading.

#### 3.2.3 2D Fillers

2D carbon cores are usually made of graphite or graphene nanoplatelets (GNPs). As for the 1D core–shell fillers based on MWCNTs, most 2D carbon@ceramic core–shell fillers are made with a SiO_2_ shell. However, contrary to what has been observed for 1D MWCNT@SiO_2_ core–shell nanofillers, 2D graphite@SiO_2_ fillers are mainly fabricated with a prior noncovalent activation, instead of a severe oxidation. For instance, Choi et al.^[^ ^61^, ^74^ ^]^ used PVP (*M*_w_ = 40 000 g mol^−1^) as a coupling agent to activate the surface of raw graphite flakes prior to the reaction with TEOS molecules. The first step consisted in the physical adsorption of PVP on graphite surface by firstly dissolving a certain amount of PVP into water, then adding raw graphite to the solution. After proper sonication and vigorous mixing, the PVP‐adsorbed graphite was filtered and rinsed with water. Then, it was redispersed in ethanol and a certain amount of NH_4_OH was introduced in the solution to catalyze the condensation of TEOS molecules at the surface of PVP‐grafted graphite. The authors reported that a uniform film of silica was grown at the surface of PVP‐grafted graphite in particular optimum conditions (60 wt% of PVP and 120 wt% of TEOS with respect to the amount of graphite). They proposed a mechanism that explains the PVP‐assisted silica‐coating method (Figure 5c). At sufficiently high pH, the PVP undergoes keto–enol tautomerization, which reveals an equilibrium between its carbanion and its enolate ion. This is theoretically on the enolate ion (or the enol) that hydrolyzed TEOS molecules react to finally form the SiO_2_ shell through condensation reactions. In their study, Choi et al.^[^ ^61^ ^]^ measured a SiO_2_ shell thickness at ≈50–60 nm on their graphite@SiO_2_ core–shell particles, via TEM images. Once introduced inside a polymer matrix (a thermoplastic polyester elastomer, TPEE), the graphite@SiO_2_ fillers provided a thermal conductivity of 2.39 W m^−1^ K^−1^ in the parallel direction, at 80 phr (44 wt%) loading, which was 32.5% lower than the value measured on TPEE‐raw graphite composites. Although the SiO_2_ shell provided electrical insulation to the composite (electrical resistivity measured at 1.00 × 10^13^ Ω cm), its rather large layer thickness retarded the phonon propagation which finally contributed to the decrease of the overall thermal conductivity. Noma et al.^[^ ^60^ ^]^ also produced graphite@SiO_2_ core–shell particles by a surfactant assisted sol‐gel method, using CTAB instead of PVP as the coupling agent. The thermal conductivity measured on their polymer‐graphite@SiO_2_ composite, using polybutylene terephthalate polyester resin (PBT) as the polymer matrix, reached 3.3 W m^−1^ K^−1^ at 22.9 vol% (≈34 wt%) loading, while the electrical resistivity was satisfyingly high (1.0 × 10^14^ Ω cm). Kim et al.^[^ ^75^ ^]^ studied the influence of various amphiphiles on the quality of fabrication of graphite@SiO_2_ core–shell particles. In their study, they compared five different coupling agents including two surfactants (sodium dodecyl sulfate or SDS, and octyphenyl ethoxylate or Triton X‐100) and three polymers (PVP, PEG, and lignin), and some combinations between them. They concluded that the best way to obtain a smooth SiO_2_ layer at the surface of the graphite flakes was by using a combination of Triton X‐100 and PEG. In another study,^[^ ^76^ ^]^ they introduced their as created graphite@SiO_2_ core–shell fillers in a TPEE matrix to form TPEE‐graphite@SiO_2_ composites. They assessed their thermal and electrical properties, and similarly to what has been previously observed by Choi et al., they found that the 45–65‐nm‐thick SiO_2_ layer on graphite@SiO_2_ caused a decrease in thermal conductivity of the composite compared to the TPEE‐raw graphite composite (20% lower, from 2.6 to 2.1 W m^−1^ K^−1^ at the same 30 vol% filler loading). However, the SiO_2_ shell provided the expected electrical insulation properties. Compared to the standard ceramic fillers studied (BN and alumina), the graphite@SiO_2_ core–shell fillers provided the best trade‐off, showing the very competitive interest of the core–shell technology for thermally conductive and electrically insulating polymer composites.

Other ceramic shells were also investigated for graphite cores. Liu et al.^[^ ^34^ ^]^ prepared graphite@Al_2_O_3_ core–shell particles through graphite surface activation by a surfactant (SDS). Subsequently, the suspension was stirred and an Al(NO_3_)_3_‐9H_2_O and NaOH aqueous solutions was added into the suspension. After obtaining graphite@Al(OH)_3_, the product was thermally treated in a tubular furnace at 600 °C for 3 h to finally obtain the expected graphite@Al_2_O_3_ core–shell particles. The latter were then added to a mixture of phthalonitrile (PN) monomers and a curing agent to obtain PN‐graphite@Al_2_O_3_ composites after thermal curing. At 20 wt% loading, the composite filled with the graphite@Al_2_O_3_ fillers was still electrically insulating (electrical resistivity of 1.33 × 10^10^ Ω cm), whereas the PN‐graphite composite was electrically conductive upon loading of 5 wt% or more. The thermal conductivity was measured at 0.7 W m^−1^ K^−1^ at 20 wt% loading, which was a little bit lower than the value measured on PN‐graphite composites but largely higher than the composites using only Al_2_O_3_ fillers. This demonstrates once again the interest of using core–shell fillers instead of standard ceramic fillers.

Graphene sheets (GS), usually obtained by exfoliating graphite flakes, are innovative carbon‐based fillers. Several authors considered using GS and GNPs as the core material of core–shell nanostructures for thermally conductive and electrically insulating polymer nanocomposites. Similarly to graphite@SiO_2_ core–shell particles, graphene@SiO_2_ core–shell nanostructures have been developed. Pu et al.^[^ ^77^ ^]^ exfoliated graphite oxide into GO sheets by ultrasonication. To facilitate the grafting of hydrolyzed TEOS molecules on the surface of the graphene sheets, an intermediate step consisting of grafting APTES at the surface of the graphene oxide sheets was conducted. The graphene@SiO_2_ core–shell particles were then obtained through a sol‐gel process using TEOS as a precursor of SiO_2_ and under basic conditions (using NH_4_OH) in an ethanol‐water solution, as described hereinbefore. Once obtained, these core–shell nanofillers were introduced in an epoxy matrix to form epoxy‐graphene@SiO_2_ nanocomposites. Thanks to the SiO_2_ layer, the epoxy‐graphene nanocomposite became electrically insulating with an electrical resistivity measured at 1.70 × 10^14^ Ω cm at 8 wt% (≈3.8 vol%). The thermal conductivity was also increased by 72%, up to 0.3 W m^−1^ K^−1^. This increase in thermal conductivity performance was explained by a modulus mismatch reduction between the filler and the matrix and by the improvement of the interfacial interaction ascribed to the SiO_2_ interlayer. Shen et al.^[^ ^78^ ^]^ prepared silica‐coated graphene nanoplatelets (GNP@SiO_2_) through the use of the cationic surfactant CTAB as the intermediate grafting site. Once the CTAB physically adsorbed onto the GNP surface after ultrasonication in an ethanol‐water solution, GNP@SiO_2_ core–shell particles were obtained via the same sol‐gel process described previously using TEOS. When dispersed in the PDMS, GNP@SiO_2_ core–shell nanofillers provided to the nanocomposite a thermal conductivity value of 0.5 W m^−1^ K^−1^ at 2 wt% (≈0.9 vol%) loading, a little higher than the value measured on PDMS nanocomposites using raw GNPs. Most interestingly, the PDMS‐GNP@SiO_2_ nanocomposites remained electrically insulating with electrical resistivity values above 10^13^ Ω cm, whereas the PDMS‐raw GNPs nanocomposites became electrically conductive upon loading of 1 wt% or more. Wang et al.^[^ ^79^ ^]^ fabricated modified GNP@SiO_2_ core–shell particles using CTAB as a primer, following the same scheme developed by Shen et al. A significant difference lies in the fact that Wang et al. mixed an organosilane (MPS) with TEOS during the sol‐gel synthesis step, thereby forming a hybrid GNP@SiO core–shell nanofiller. Once introduced in an epoxy matrix, GNP@SiO increased the thermal conductivity of the epoxy‐GNP@SiO nanocomposite up to 0.8 W m^−1^ K^−1^ at 1 wt% (≈0.46 vol%) while remaining electrically insulating (electrical resistivity of 3.0 × 10^12^ Ω cm). Interestingly, when Ag nanoparticles were grown at the surface of the GNP@SiO core–shell nanostructures, the corresponding epoxy‐GNP@SiO@Ag nanocomposite exhibited an even higher thermal conductivity value of 1.03 W m^−1^ K^−1^ at the same loading ratio of 1 wt%, while remaining electrically insulating.

As for graphite cores, other ceramic shells such as Al_2_O_3_ were investigated for graphene sheets and nanoplatelets. Qian et al.^[^ ^80^ ^]^ coated graphene sheets with alumina by electrostatic self‐assembly. More precisely, Al_2_O_3_ nanoparticles were firstly modified with an aminosilane (APTES) which rendered the Al_2_O_3_ surfaces positively charged. At the same time, graphene oxide was prepared by a modified Hummers method, which is a chemical exfoliation method. Graphene oxide is believed to be negatively charged due to the hydroxyl, carboxyl, and epoxide functional groups on its surface. The modified Al_2_O_3_ nanoparticles were then assembled with graphene oxide to form GO@Al_2_O_3_ core–shell particles by favorable electrostatic interactions. The GO@Al_2_O_3_ particles were eventually chemically reduced with hydrazine to obtain the expected GS@Al_2_O_3_ core–shell nanofillers. Once added into a PVDF matrix to form PVDF‐GS@Al_2_O_3_ nanocomposites, thermal and electrical properties were investigated. At 40 wt% loading (≈34 vol%), thermal conductivity increased as the amount of Al_2_O_3_ at the surface of GS increased until an optimum was reached using a mass ratio of GS to Al_2_O_3_ equal to 1:20. At this point, electrical resistivity was measured at 4.10 × 10^14^ Ω cm and thermal conductivity at 0.59 W m^−1^ K^−1^. The Al_2_O_3_ interlayer contributed to lower the interfacial thermal resistance through the lowering of the acoustic mismatch between GS and PVDF. However, with more Al_2_O_3_ at the surface of GS, the electrical resistivity was increased but thermal conductivity decayed due to the low intrinsic thermal conductivity of Al_2_O_3_. Sun et al.^[^ ^81^ ^]^ fabricated GNP@Al_2_O_3_ using two comparative methods. Their GNPs were obtained by thermal exfoliation of graphite oxide followed by annealing in an argon atmosphere. The first approach consisted in using supercritical carbon dioxide in the presence of Al(NO_3_)_3_‐9H_2_O precursor in a high‐pressure autoclave. After reaction, the resultant product was calcined at 600 °C for 3 h in an inert atmosphere to obtain GNP‐sc@Al_2_O_3_ core–shell nanostructures with uniformly dispersed Al_2_O_3_ nanoparticles coated onto the GNPs. The second approach consisted in using an aqueous buffer solution of formic acid and ammonium formate (pH = 4.4). GNPs mildly treated with HNO_3_ were then added to the buffer solution with addition of Al_2_(SO_4_)_3_‐18H_2_O as a precursor. The product was calcinated in the same conditions as that for the first approach and GNP‐bs@Al_2_O_3_ core–shell nanostructures with uniform Al_2_O_3_ nanolayers were retrieved. The two species of GNP@Al_2_O_3_ prepared in different conditions were finally incorporated in an epoxy matrix to form epoxy‐GNP@Al_2_O_3_ nanocomposites. For comparison, epoxy‐based composites loaded with other commercial fillers were also prepared. At 12 wt% filler loading, the epoxy‐based nanocomposite using GNP‐bs@Al_2_O_3_ nanofillers exhibited the best thermal conductivity compared to other fillers such as Al_2_O_3_, hBN, MWCNTs, and raw GNPs. The thermal conductivity values were also superior to that measured for epoxy‐GNP‐sc@Al_2_O_3_ nanocomposites, showing that a uniform Al_2_O_3_ nanolayer is more efficient in reducing interfacial thermal resistance than several uniformly dispersed Al_2_O_3_ nanoparticles. For epoxy‐GNP‐bs@Al_2_O_3_ nanocomposites, the thermal conductivity reached 1.49 W m^−1^ K^−1^ at 12 wt% (≈6.6 vol%) loading. Moreover, the Al_2_O_3_ nanolayer made the epoxy‐GNP@Al_2_O_3_ nanocomposite electrically insulating with a low electrical conductivity measured value of 6.70 × 10^−9^ S m^−1^. Therefore, using a GNP@Al_2_O_3_ core–shell nanofiller not only reduced the electrical conductivity, which is needed for electronic packaging applications, but also improved the thermal dissipation performance by increasing the thermal conductivity of the nanocomposite compared to other standard ceramic fillers. The thermal conductivities and electrical resistivities of polymer nanocomposites made with carbon‐based core–shell nanofillers are summarized in **Table** **2**.

**Table 2:** Thermal conductivities and electrical resistivities of polymer nanocomposites made with carbon‐based core–shell nanofillers.

Polymer matrix	Core–shell filler	Surface activation of the core	Nanocomposite processing route	Loading ratio [wt%‐vol%]	Thermal conductivity of the pure polymer matrix [W m^−1^ K^−1^]	Thermal conductivity of the nanocomposite [W m^−1^ K^−1^]	Electrical resistivity [Ω cm]	Ref.
Epoxy	MWCNT@SiO_2_	Acid treatment (HNO_3_/H_2_SO_4_)	Solvent	1.0‐0.5	0.14	0.24	6.90 × 10^16^	[31]
PU				1.0‐0.58	0.18	0.31	1.00 × 10^14^	[65]
PVDF	MWCNT@SiO_2_ *(hybridized with hBN)*		Solvent	25 (mass ratio MWCNT@SiO_2_:hBN = 1:4)	0.22	1.51	3.50 × 10^12^	[62]
Epoxy	MWCNT@SiO_2_‐*g*‐BMI		Blending	1.25‐0.62	0.20	0.45	2.90 × 10^15^	[67]
Epoxy	MWCNT@SiO_2_	CTAB (surfactant)		1.0‐0.5	0.19	0.55	2.00 × 10^12^	[69]

PI	MWCNT@BN	Acid treatment (HNO_3_)	Solvent	3.00‐1.98	0.19	0.39	7.69 × 10^9^	[70]

Epoxy	MWCNT@MgO	Acid treatment (HNO_3_/H_2_SO_4_)	Solvent	2.0‐1.0	0.19	0.36	2.40 × 10^16^	[71]

PVDF	MWCNT@BN/SiO_2_	–	Solvent	30‐*n.a*.	0.14	1.56	*n.a*.	[66]

SR (silicon rubber)	CF@BN	Cooling precipitation (polyacrylic acid/H_3_BO_3 _/C_3_H_6_N_6_) Thermal treatment	Blending	20‐*n.a*.	*n.a*.	12.06	5 × 10^9^	[73]

TPEE	Graphite@SiO_2_	PVP (amphiphile polymer)	Blending	44‐*n.a*.	0.20	2.39	1.00 × 10^13^	[61]
PBT		CTAB (surfactant)		34.0‐22.9	*n.a*.	3.3	1.0 × 10^14^	[60]
TPEE		Triton X‐100 / PEG (polymers)		*n.a*.‐30	0.25	2.1	>10^11^	[76]

Epoxy	Graphene@SiO_2_	APTES silanization (after graphite exfoliation)	Solvent	8.0‐3.8	0.17	0.3	1.70 × 10^14^	[77]
PDMS	GNP@SiO_2_	CTAB (surfactant)		2.0‐0.9	0.20	0.5	>10^13^	[78]
Epoxy	GNP@SiO		Blending	1.0‐0.46	0.18	0.8	3.0 × 10^12^	[79]

PN (phthalonitrile)	Graphite@Al_2_O_3_	SDS (surfactant)	Solvent	20‐*n.a*.	0.21	0.7	1.33 × 10^10^	[34]
PVDF	GS@Al_2_O_3_	Chemical exfoliation‐electrostatic interactions		40‐34	0.20	0.59	4.10 × 10^14^	[80]
Epoxy	GNP@Al_2_O_3_	Exfoliation of graphite oxide, acid treatment		12.0‐6.6	0.22	1.49	>10^10^	[81]

### 3.3 Metal@Ceramic Fillers

**Figure 6:**
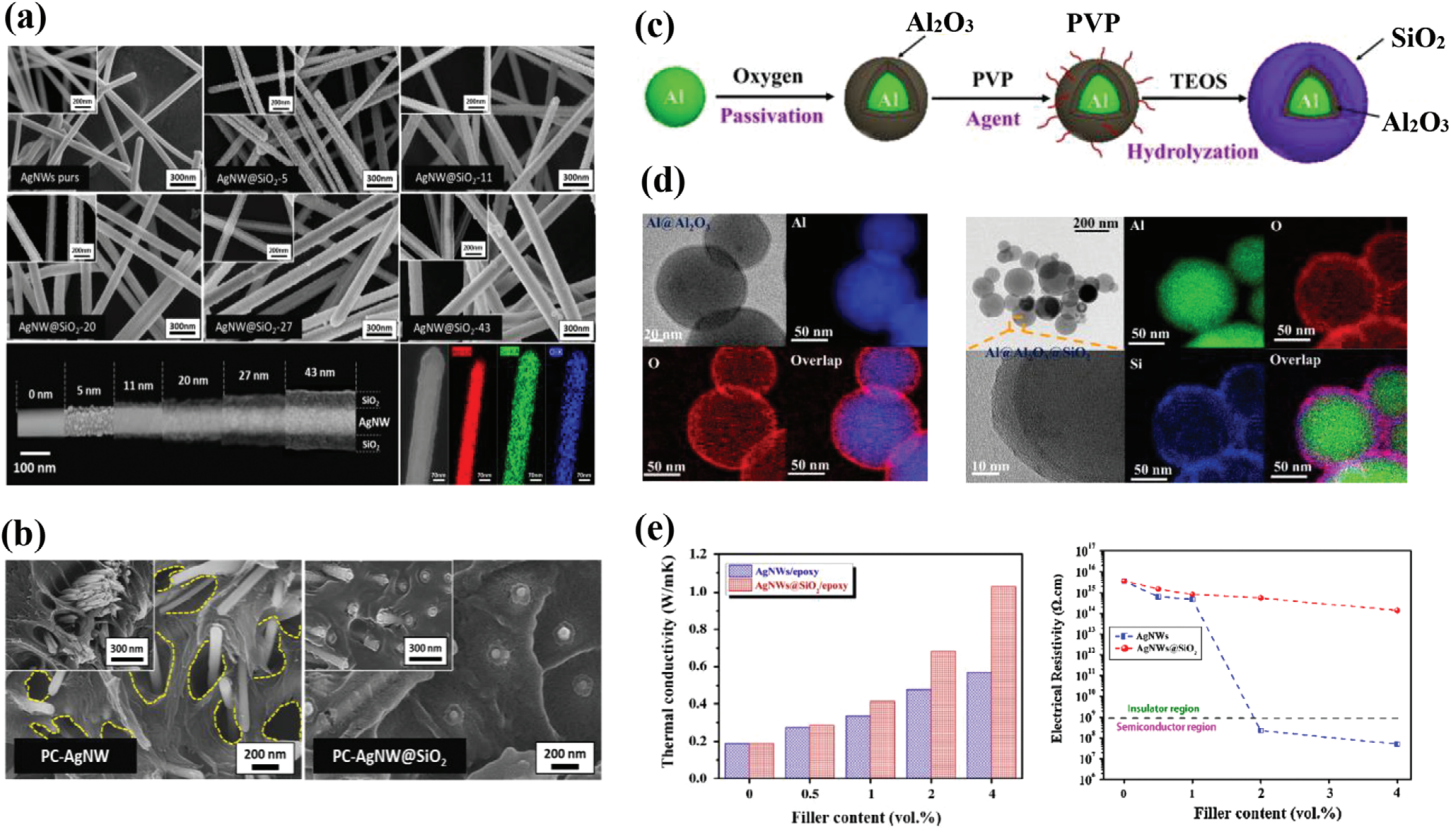
a) SEM images of AgNWs@SiO_2_ core–shell nanostructures at various thicknesses of silica. Reproduced with permission.^[^ ^82^ ^]^ Copyright 2024, Elsevier. b) SEM images of PC‐AgNW and PC‐AgNWs@SiO_2_ nanocomposites. c) Schematic illustration of the core–shell formation of Al@Al_2_O_3_@SiO_2_ particles.^[^ ^83^ ^]^ d) TEM images of Al@Al_2_O_3_ and Al@Al_2_O_3_@SiO_2_ core–shell particles and its multilayer coating structure.^[^ ^83^ ^]^ e) Filler content dependence of thermal conductivity and volume electrical resistivity for systems composed of AgNWs and AgNWs@SiO_2_. Reproduced with permission.^[^ ^84^ ^]^ Copyright 2014, Elsevier.

The last category of core–shell nanofillers discussed here features nanostructures with a metallic core. Metallic fillers generally exhibit fewer defects than carbonaceous fillers, and their intrinsic thermal conductivities are experimentally comparable, if not superior (at least for the most conductive metals, such as copper and silver). Selected examples of such structures are highlighted in **Figure** **6**.

#### 3.3.1 0D Fillers

Among metal@ceramic fillers, the most straightforward to implement are those in which the insulating shell is the oxide obtained from the metal constituting the core, usually through self‐passivation. For Al cores, the encapsulating shell is therefore made of its most common oxide, Al_2_O_3_, to produce Al@Al_2_O_3_ core–shell particles. Mao et al.^[^ ^85^ ^]^ fabricated several Al@Al_2_O_3_ core–shell fillers with various Al_2_O_3_ layer thickness by passivating the Al core in a furnace set at different temperatures ranging from 200 to 600 °C, for 8 h under an air atmosphere. While there was almost no Al_2_O_3_ at the surface of the Al cores under 300 °C, a thin Al_2_O_3_ layer began to grow at 400 °C and it thickened with increasing temperature (4, 9, and 11 nm for 400, 500, and 600 °C, respectively). Then, the authors incorporated these Al@Al_2_O_3_ core–shell particles in an epoxy matrix to form epoxy‐Al@Al_2_O_3_ nanocomposites. At 60 wt% (≈37 vol%) loading, the electrical resistivity of the nanocomposite filled with Al@Al_2_O_3_ nanofillers with a 9‐nm‐thick Al_2_O_3_ nanolayer was measured at 4.50 × 10^13^ Ω cm. The thermal conductivity progressively decreased with increasing Al_2_O_3_ thickness, but was still satisfying with a 0.92 W m^−1^ K^−1^ measured value. Xu et al.^[^ ^83^ ^]^ fabricated double core–shell structured Al@Al_2_O_3_@SiO_2_ particles through a prior self‐passivation of the Al core to form an Al@Al_2_O_3_ intermediate, followed by the addition of PVP in ethanol to activate the surface of Al_2_O_3_. TEOS, NH_4_OH and water were finally added to build the SiO_2_ nanolayer on top of the Al_2_O_3_ layer by a sol‐gel process. Once introduced in an epoxy matrix up to a 40 wt% loading, the Al@Al_2_O_3_@SiO_2_ core–shell nanofillers increased the thermal conductivity of the epoxy‐ Al@Al_2_O_3_@SiO_2_ nanocomposite up to 0.53 W m^−1^ K^−1^ while maintaining its electrical insulation with an electrical resistivity measured at 1.00 × 10^15^ Ω cm. Based on the same approach, Zhou et al.^[^ ^86^, ^87^ ^]^ constructed Al@Al_2_O_3_@SiO_2_ core–shell particles in spherical and fiber form (1D). The two kinds of fillers were then uniformly dispersed into a PI matrix at different volume proportions for a total loading of 50 vol%. While the lowest thermal conductivity value was obtained with 100% of spherical Al@Al_2_O_3_@SiO_2_ (≈2 W m^−1^ K^−1^), the highest value was obtained with a mix of 25% spherical and 75% Al@Al_2_O_3_@SiO_2_ in fiber form (15.2 W m^−1^ K^−1^). Zhou et al.^[^ ^88^ ^]^ also prepared double core–shell structured particles composed of Al as the core and two layers of Al_2_O_3_ produced by applying different temperatures of calcination under nitrogen atmosphere. This synthesis allows forming an amorphous and a crystalline layer of Al_2_O_3_ that are denoted nc‐Al_2_O_3_ and c‐Al_2_O_3_, respectively. The double core–shell Al@nc‐Al_2_O_3_@c‐Al_2_O_3_ particles were then introduced in a PVDF matrix. The dielectric and thermal properties of the PVDF‐Al@nc‐Al_2_O_3_@c‐Al_2_O_3_ nanocomposite were improved due to the enhanced interfacial polarization that is induced by preventing the long‐range migration of electrons within the different interfaces. Li et al.^[^ ^89^ ^]^ fabricated Ni@NiO core–shell particles by self‐passivation of the Ni core (1h at 550 °C under air atmosphere). Once added to a PVDF matrix, they obtained a thermal conductivity of 1.08 W m^−1^ K^−1^ at 70 wt% (≈32 vol%) loading, while conserving a good electrical insulation with an electrical conductivity lower than 10^−9^ S cm^−1^ thanks to the NiO insulating layer. Yao et al.^[^ ^90^ ^]^ used Zn@ZnO core–shell particles, produced by calcinating the Zn cores at 400 °C for 3 h under air atmosphere, and polymerized polystyrene (PS) at their surface to make Zn@ZnO@PS nanoparticles. These nanofillers were then added to a PVDF matrix to produce PVDF‐Zn@ZnO@PS nanocomposites. At 40 wt% (≈14 vol%) loading, they obtained a thermal conductivity of 0.54 W m^−1^ K^−1^ for an electrical resistivity of 3.00 × 10^13^ Ω cm.

0D metal@ceramic core–shell fillers can also be made with a shell that is unrelated to its core. Copper (Cu), which possesses the second‐highest thermal conductivity of all metals (behind silver), is often used as a core material, in particular because it is about a hundred times cheaper than silver. Wang et al.^[^ ^33^ ^]^ fabricated Cu@Al_2_O_3_ core–shell particles through a prior activation of the Cu core with PVP followed by a sol‐gel reaction using Al_2_(SO_4_)_3_‐18H_2_O as a Al_2_O_3_ precursor. The Al_2_O_3_ nanolayer is then obtained after a heat treatment at 250 °C for 2 h under air atmosphere. Once added in an epoxy matrix to form an epoxy‐Cu@Al_2_O_3_ nanocomposite, the Cu@Al_2_O_3_ core–shell nanofillers allowed the nanocomposite to remain electrically insulating with an electrical resistivity measured at 2.30 × 10^13^ Ω cm for a 10 vol% loading, thanks to its Al_2_O_3_ insulating shell. At the same loading ratio, the thermal conductivity of the nanocomposite was measured at 1.32 W m^−1^ K^−1^, a value nearly seven times higher than that of the epoxy resin. Wang et al.^[^ ^91^ ^]^ fabricated Cu@BaTiO_3_ core–shell particles in a similar fashion, with a prior Cu activation by PVP followed by a sol‐gel reaction of a BaTiO_3_ precursor. Upon addition of the Cu@BaTiO_3_ core–shell fillers in an epoxy matrix, the authors measured a thermal conductivity of 1.09 W m^−1^ K^−1^ at 10 vol% loading for a retained electrical insulation with a corresponding electrical resistivity of 7.90 × 10^13^ Ω cm. Zhou et al.^[^ ^92^ ^]^ synthesized core–shell structures by coating Cu particles with a thin oxidation layer of CuO synthesized by calcination under air atmosphere. The Cu@CuO core–shell particles were introduced in a PVDF matrix in order to quantify the dielectric and the thermal properties. Due to the calcination treatment, the CuO shell hindered the long‐distance electron migration and reduced the thermal resistance at the interface. Consequently, the final nanocomposite PVDF‐Cu@CuO exhibited improved dielectric performance and enhanced thermal conductivity. Lee et al.^[^ ^93^ ^]^ prepared Cu@BN core–shell particles through thermal annealing of Cu powder coated with melamine diborate at 1000 °C for 3 h to produce a layer of BN. Then polysilazane was finally coated (Cu@BN@PSZ) to improve the filler interaction with the epoxy matrix. At 60 wt% loading, the epoxy‐Cu@BN@PSZ composite exhibited a thermal conductivity of 3.47 W m^−1^ K^−1^ and an electrically insulating behavior, with a reported electrical resistivity of 10^11^ Ω cm. Zhou et al.^[^ ^32^ ^]^ synthesized Ag@SiO_2_ core–shell particles and incorporated them in a PI matrix to form PI‐Ag@SiO_2_ nanocomposites. At 50 vol% loading, they obtained a thermal conductivity of 7.88 W m^−1^ K^−1^ while the SiO_2_ layer retained the electrical insulation of the nanocomposite (electrical resistivity of 3.40 × 10^13^ Ω cm).

#### 3.3.2 1D Fillers

Other authors have focused on nanofillers with high‐aspect‐ratio geometries to limit the overall loading rate, such as 1D metal nanowires. Zhou et al.^[^ ^36^ ^]^ synthesized copper nanowires (CuNWs) and coated them with a layer of boron nitride using boric acid and urea in solution, followed by a thermal treatment in a furnace at 900 °C for 3 h under a NH_3_ atmosphere. The resulting CuNW@BN core–shell nanowires were then incorporated in a PI matrix to form PI‐CuNW@BN nancomposites. At 20 vol% loading, the thermal conductivity was measured at 4.12 W m^−1^ K^−1^ while the insulating BN layer improved the electrical resistivity up to 4.80 × 10^13^ Ω cm. In a similar fashion, the same authors^[^ ^94^ ^]^ synthesized silver nanowires (AgNWs) coated with a thin BN layer (≈9 nm thickness) to form AgNW@BN core–shell nanowires. At 20 vol% loading in a PI matrix, they measured a thermal conductivity of 4.30 W m^−1^ K^−1^, which is slightly higher than the one measured for CuNW cores. The BN nanolayer enhanced the dispersion of the AgNWs in the PI matrix as well as the interfacial interaction between AgNWs and the PI matrix. The BN nanolayer is also believed to reduce the modulus mismatch between AgNWs and the PI matrix, thereby decreasing the thermal interfacial resistance. This participates in improving the overall thermal conductivity of the nanocomposite. The BN layer allowed the nanocomposite to remain electrically insulating with an electrical resistivity measured at 3.90 × 10^13^ Ω cm. The dielectric properties of the PI‐AgNW@BN nanocomposites were in line with expectations for heat dissipation in microelectronics applications. Ahn et al.^[^ ^95^ ^]^ synthesized CuNW@TiO_2_ core–shell nanowires with a thin TiO_2_ shell of ≈3 nm in thickness. Added in an epoxy matrix, the core–shell nanofillers improved the thermal conductivity of the nanocomposite up to 1.12 W m^−1^ K^−1^ at 15 wt% (≈2 vol%) loading. This was higher than the values measured for the nanocomposites using pure CuNWs as nanofillers, and the given explanation was related to a decrease of the modulus mismatch between nanofillers and the epoxy matrix thanks to the thin TiO_2_ nanolayer. The latter also participated in the reduction of the electrical conductivity below 7.0 × 10^−8^ S cm^−1^. By comparison, Jiang et al.^[^ ^96^ ^]^ fabricated AgNW@TiO_2_ core–shell nanowires through a sol‐gel process using tetrabutyl‐titanate (TBOT) as a precursor, and retrieved AgNW@TiO_2_ nanowires with a TiO_2_ shell of ≈30 nm in thickness. At first, they thought that the TiO_2_ shell would further improve the thermal conductivity of their epoxy‐AgNW@TiO_2_ nanocomposite compared to a SiO_2_ shell because of an intrinsic thermal conductivity in favor of TiO_2_ (1 vs 10 W m^−1^ K^−1^ for SiO_2_ and TiO_2_, respectively). However, they observed that the epoxy‐AgNW@TiO_2_ nanocomposites exhibited lower thermal conductivities than the epoxy‐AgNW ones (0.8 vs 0.9 W m^−1^ K^−1^, respectively, at 4 vol% loading, for long nanowires), although the dispersion and interfacial interaction was improved thanks to the TiO_2_ layer. This was explained by the increase of the modulus mismatch between AgNW and the epoxy matrix caused by the 30‐nm‐thick TiO_2_ layer. Indeed, the 30‐nm‐thick TiO_2_ layer used in the study possessed a higher elastic modulus than the 3‐nm‐thick TiO_2_ layer used in the study of Ahn et al. previously described. The higher elastic modulus mismatch enhanced phonon scattering at the epoxy–AgNW interface, thereby increasing the interfacial thermal resistance, and decreasing the thermal conductivity of the nanocomposite. On another aspect, the TiO_2_ nanolayer helped to maintain the electrical resistivity of the nanocomposite up to 7.10 × 10^12^ Ω cm at 4 vol%, what was not achievable with pure AgNWs. The same team (Chen et al.^[^ ^84^ ^]^) also fabricated AgNW@SiO_2_ core–shell nanowires through a sol‐gel process in ethanol and water using TEOS as a precursor and NH_4_OH as a basic catalyst. With this preparation method, they retrieved AgNW@SiO_2_ core–shell nanowires with a uniform SiO_2_ shell of about 20–25 nm in thickness. When added to an epoxy matrix to form epoxy‐AgNW@SiO_2_ nanocomposites, the AgNW@SiO_2_ core–shell nanowires improved the thermal conductivity of the epoxy up to 1.03 W m^−1^ K^−1^ at 4 vol% loading, which was significantly higher than the value measured on nanocomposites using pure AgNWs (0.57 W m^−1^ K^−1^). The SiO_2_ nanolayer coated on the AgNWs in AgNW@SiO_2_ provided strong enhancements on dispersion of the AgNWs in the epoxy matrix and improved the interfacial interaction between AgNWs and the epoxy matrix. This is believed to help improve the creation of performant thermal pathways across the nanocomposite and reduce the phonon scattering at the interface. The SiO_2_ nanolayer is also believed to act as an intermediate layer to alleviate the modulus mismatch between the soft epoxy matrix and the stiff AgNWs, thereby reducing the interfacial thermal resistance and participating in the improvement of the overall thermal conductivity of the nanocomposite. The SiO_2_ nanolayer also maintained the electrical insulation of the nanocomposite with electrical resistivity measured at 1.40 × 10^14^ Ω cm at 4 vol% loading, where the electrical insulation of epoxy‐AgNWs nanocomposites broke before 2 vol% loading. The dielectric properties of the epoxy‐AgNW@SiO_2_ nanocomposites were also in line with expectations for its use in integrated circuits for the electronic packaging industry. In one of our previous work,^[^ ^82^ ^]^ AgNW@SiO_2_ core–shell nanowires were also synthesized through a similar sol‐gel process with fine tuning of the SiO_2_ thickness (from 5 to 43 nm) to decipher the influence of the shell thickness on thermal and electrical performances of a polycarbonate (PC) nanocomposite (PC‐AgNW@SiO_2_). It was found that uniform and conformal SiO_2_ nanolayers could be grown at the surface of AgNWs and that the thickness could be controlled and tailored through the control of the sol‐gel experimental parameters. As in other studies, it was found that the SiO_2_ shell improved both the dispersion of the AgNWs in the PC matrix and the interfacial interaction (no more AgNW bundles and no more voids between the AgNWs and the PC matrix). It was also found that an optimum SiO_2_ layer thickness for an optimum thermal conductivity could be determined and that it lay around 20 nm. For a 3 vol% loading, the PC‐AgNW@SiO_2_ nanocomposite with a 20‐nm‐thick SiO_2_ shell exhibited an optimum thermal conductivity of 2.08 W m^−1^ K^−1^. For thicker layers, the thermal conductivity of the nanocomposite decreased progressively, as observed in other systems found in the literature.^[^ ^51^, ^61^, ^76^, ^97^ ^]^ This reversing trend in thermal conductivity enhancement suggests that the low intrinsic thermal conductivity of SiO_2_ and the increasing modulus mismatch associated with an increasing layer thickness outweigh the good dispersion and the enhanced filler–matrix cohesion. Concerning electrical resistivity, it was only at thicknesses of 20 nm and above that the SiO_2_ coating was fully effective with a value measured at 1.13 × 10^12^ Ω cm for the 20‐nm‐SiO_2_ thick PC‐AgNW@SiO_2_ nanocomposite. Dielectric properties were in line with expectations for battery casing and electronics applications. One of the most important advantages of this PC‐AgNW@SiO_2_ nanocomposite is its 3D printability by fused deposition modeling (FDM). Moreover, its thermal conductivity was significantly increased in the printing direction, up to 3.48 W m^−1^ K^−1^ while retaining good electrical insulation. Kim et al.^[^ ^97^ ^]^ fabricated CuNW@SiO_2_ core–shell nanowires by a sol‐gel reaction followed by sintering at 200 °C for 1 h in a N_2_ atmosphere. Although the CuNW@SiO_2_ nanowires did not seem to be uniformly coated by an SiO_2_ layer, they managed to improve the thermal conductivity of an ETDS (epoxy‐terminated dimethylsiloxane) composite up to 1.1 W m^−1^ K^−1^ at 15 wt% (≈2 vol%) loading, better than raw CuNWs. It was also observed that with an increase of the SiO_2_ thickness, the thermal conductivity tended to decrease due to its low intrinsic thermal conductivity and to SiO_2_ aggregated layers (causing phonon scattering between layers). Nonetheless, the SiO_2_ shell did maintain the electrical conductivity of the nanocomposite below 10^−9^ S cm^−1^. Zhang et al.^[^ ^98^ ^]^ prepared AgNW@ZnO core–shell nanowires by the precipitation method using Zn(NO_3_)_2_‐6H_2_O as a precursor. Even though the ZnO coating was quite rough and not really uniform, they managed to slightly increase the thermal conductivity of an epoxy‐AgNW@ZnO nanocomposite up to 0.77 W m^−1^ K^−1^ at 8 wt% (≈0.9 vol%) loading, corresponding to a 22% enhancement over the epoxy‐AgNWs nanocomposite. Thanks to the ZnO layer, the electrical resistivity of the epoxy‐AgNW@ZnO nanocomposite remained above 10^13^ Ω cm over the whole range of tested loading ratios, while the electrical resistivity of the epoxy‐AgNW nanocomposite dropped sooner.

Other authors decided to mix AgNW@SiO_2_ with other fillers such as GNPs^[^ ^99^ ^]^ to take advantage of synergistic hybrid networks. In this study, Yang et al. found that with the addition of 2.7 vol% of GNPs to an epoxy‐AgNW@SiO_2_ nanocomposite already loaded at 0.8 vol%, the thermal conductivity jumped from 0.44 to 1.09 W m^−1^ K^−1^ while the electrical insulation remained satisfying. Zhuang et al.^[^ ^100^ ^]^ found that the addition of graphite@SiO_2_ to a PI‐AgNW@SiO_2_ nanocomposite was beneficial to the thermal conductivity of the nanocomposite. With loadings of 15 vol% of graphite@SiO_2_ and 5 vol% of AgNW@SiO_2_, the thermal conductivity was measured at 3.21 W m^−1^ K^−1^ whereas the thermal conductivity of the 20 vol% AgNW@SiO_2_ loaded nanocomposite was measured at only 0.74 W m^−1^ K^−1^. The metal@ceramic core–shell filled nanocomposites along with their thermal conductivities and electrical resistivities are summarized in **Table** **3**.

**Table 3:** Thermal conductivities and electrical resistivities of polymer nanocomposites made with metal@ceramic core–shell nanofillers.

Polymer matrix	Core–shell filler	Surface activation of the core	Nanocomposite processing route	Loading ratio [wt%‐vol%]	Thermal conductivity of the pure polymer matrix [W m^−1^ K^−1^]	Thermal conductivity of the nanocomposite [W m^−1^ K^−1^]	Electrical resistivity [Ω cm]	Ref.
Epoxy	Al@Al_2_O_3_	Self‐passivation	Blending	60‐37	0.22	0.92	4.50 × 10^13^	[85]
Epoxy	Al@Al_2_O_3_@SiO_2_	Self‐passivation + PVP		40‐*n.a*.	0.17	0.53	1.00 × 10^15^	[83]
PI	Al@Al_2_O_3_@SiO_2_			*n.a*.‐50	*n.a*.	2.00	*n.a*.	[87]
PVDF	Ni@NiO	Self‐passivation	Solvent	70‐32	*n.a*.	1.08	*n.a*.	[89]
PVDF	Zn@ZnO@PS	Self‐passivation + polymerization		40‐14	0.20	0.54	3.00 × 10^13^	[90]
Epoxy	Cu@Al_2_O_3_	PVP	Blending	*n.a*.‐10	0.19	1.32	2.30 × 10^13^	[33]
	Cu@BaTiO_3_				0.16	1.09	7.90 × 10^13^	[91]
	Cu@BN@PSZ	Melamine diborate coating + Thermal annealing		60‐*n.a*.	0.20	3.47	10^11^	[93]
PI	Ag@SiO_2_	PVP		*n.a*.‐50	*n.a*.	7.88	3.40 × 10^13^	[32]
PI	CuNW@BN	Boric acid + urea	*n.a*.	*n.a*.‐20	0.18	4.12	4.80 × 10^13^	[36]
	AgNW@BN		*n.a*.		0.19	4.33	3.90 × 10^13^	[94]
Epoxy	CuNW@TiO_2_	Hydrazine in (NaOH + EDA)	Blending	15‐2	0.20	1.12	*n.a*.	[95]
Epoxy	AgNW@TiO_2_	Residual PVP	Blending	*n.a*.‐4	0.19	0.8	7.10 × 10^12^	[96]
	AgNW@SiO_2_					1.03	1.40 × 10^14^	[84]
PC	AgNW@SiO_2_	Residual PVP	Solvent	21.5‐3	0.23	3.48	1.13 × 10^12^	[82]
Epoxy	CuNW@SiO_2_	*n.a*.	Blending	15‐2	0.19	1.10	*n.a*.	[97]
Epoxy	AgNW@ZnO	*n.a*.	Solvent	8‐0.9	0.18	0.77	>10^13^	[98]

### 3.4 Core@Polymer Fillers

**Figure 7:**
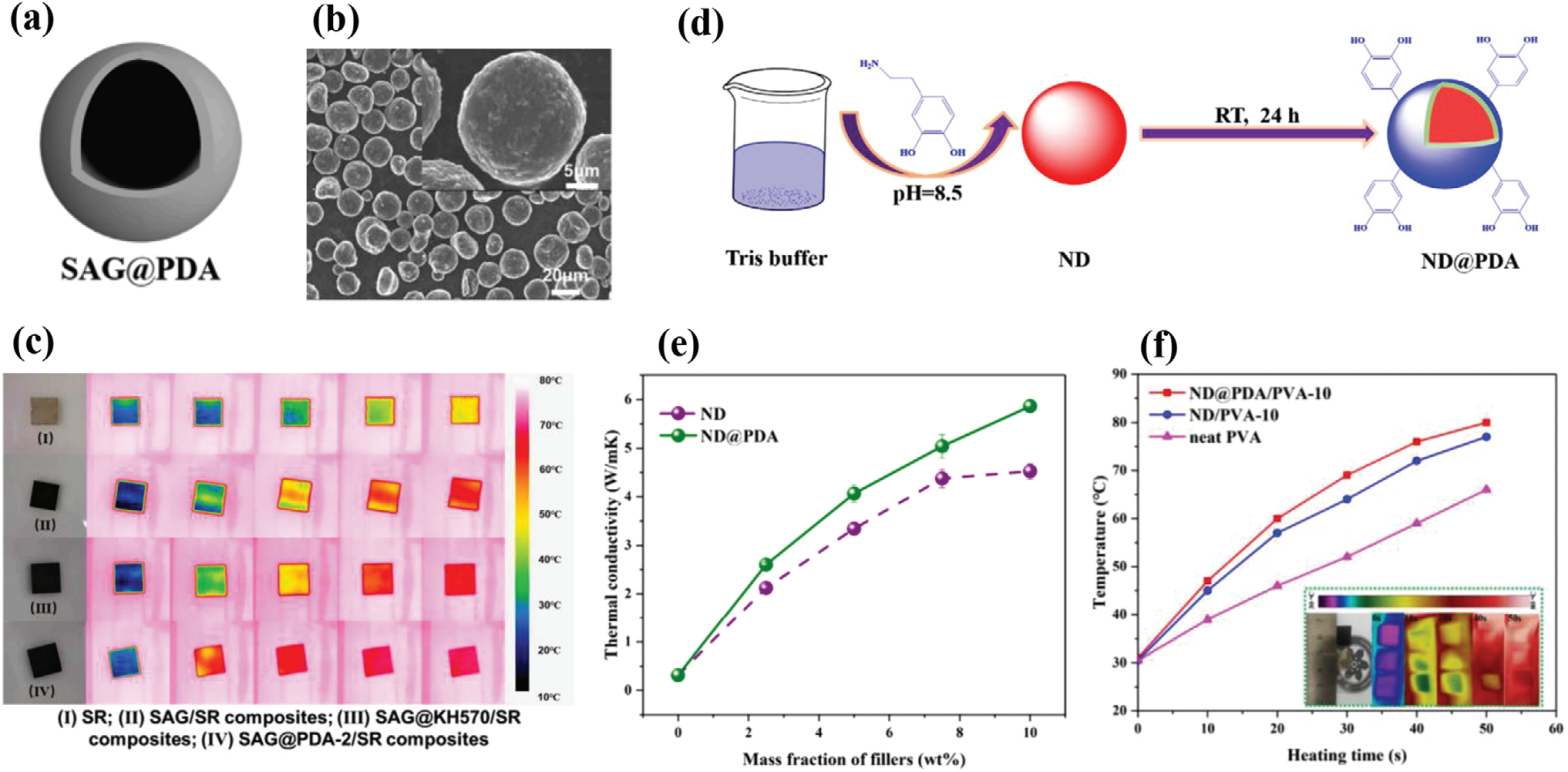
Polymer shell can improve significantly the performances of nanocomposites. a) Schematic representation and b) SEM image of polydopamine coated on spherical artificial graphite particles (SAG@PDA).^[^ ^105^ ^]^ c) Infrared images of the pure SR (silicone rubber matrix), SAG/SR and advanced SAG@SR core–shell composites during heating. Reproduced with permission.^[^ ^105^ ^]^ Copyright 2023, Elsevier. d) Surface modification scheme of nanodiamonds particles via dopamine.^[^ ^102^ ^]^ e) Thermal conductivity of ND/PVA and ND@PDA/PVA nanocomposites with different filler loadings.^[^ ^102^ ^]^ f) Infrared thermal images of surface temperature variations as a function of heating time.^[^ ^102^ ^]^ (d–f) Reproduced with permission.^[^ ^102^ ^]^ Copyright 2020, Elsevier.

Another more specific family of core–shell nanofillers concerns heterostructures with a polymer shell. The most widely used polymer shell is PDA because it has demonstrated outstanding adhesion ability and compatibility toward a variety of substrates including ceramics, carbons, and metals. For instance, Zhang et al.^[^ ^101^ ^]^ synthesized hBN@PDA core–shell nanoparticles and incorporated them in a SR matrix to make SR‐hBN@PDA nanocomposites. At 30 wt% loading, they obtained a thermal conductivity of 0.95 W m^−1^ K^−1^, higher than the nanocomposite filled with raw hBN. This improvement was explained by the fact that PDA acted as a compatibilizer between hBN and the SR matrix, thus improving the interfacial cohesion between both. The electrical resistivity was maintained and measured at 2.50 × 10^11^ Ω cm. In a similar fashion, Nan et al.^[^ ^102^ ^]^ synthesized a PDA coating at the surface of nanodiamonds (ND) to create ND@PDA core–shell nanoparticles. One incorporated in a PVA matrix, the thermal conductivity of the resulting PVA‐ND@PDA nanocomposite thin film was measured at 5.86 W m^−1^ K^−1^ at a 10 wt% loading. It was observed that the interface between the nanodiamonds and the PVA matrix was improved thanks to the PDA layer. The electrical insulation was preserved with an electrical resistivity value measured at 6.10 × 10^15^ Ω cm. Sang et al.^[^ ^103^ ^]^ prepared BNNS@PDA core–shell microspheres functionalized with hexanediol diacrylate (HDDA) by emulsification that were incorporated into a PVDF matrix. At 25 wt% of BNNS loading, the thermal conductivity reached 3.20 W m^−1^ K^−1^, which is a significant increase compared to the pure PVDF matrix (0.22 W m^−1^ K^−1^). Wang et al.^[^ ^104^ ^]^ prepared Si@SiO_2_@PDA core–shell microspheres by a thermal oxidation of raw Si particles (Si@SiO_2_), followed by the addition of dopamine‐HCl. At 33.8 vol% loading, the epoxy‐Si@SiO_2_@PDA composite exhibited a moderate thermal conductivity of 0.891 W m^−1^ K^−1^ and an electrically insulating behavior with an electrical resistivity of ≈10^18^ Ω cm. Kong et al.^[^ ^105^ ^]^ successfully synthesized PDA at the surface of spherical graphite (SAG) to form SAG@PDA core–shell particles. When added to a silicon rubber matrix at a 76 vol% loading, the thermal conductivity of the resulting composite was measured at 1.76 W m^−1^ K^−1^, unfortunately no electrical resistivity data was provided. Yuan et al.^[^ ^106^ ^]^ synthesized CuNW@PDA nanowires with various PDA thicknesses ranging from 25 to 100 nm. One added in an epoxy matrix, it was observed that the nanofiller with the lowest PDA thickness (25 nm) provided the best thermal conductivity to the epoxy‐CuNW@PDA nanocomposite (2.87 W m^−1^ K^−1^ at 3 vol% loading). The thicker the PDA layer, the lower the thermal conductivity. Indeed, although the PDA layer improved the interfacial interaction between the epoxy matrix and the CuNW nanofiller (also observed with the mechanical properties), the PDA layer is almost thermally insulating. A thicker PDA layer would thus lead to lower thermal conductivities. Thanks to the PDA layer, the electrical resistivity of the nanocomposite remained high enough, with values measured above 10^14^ Ω cm. Several core@polymer systems presented hereinbefore are illustrated in **Figure** **7**.

### 3.5 Most Targeted Industrial Applications

**Figure 8:**
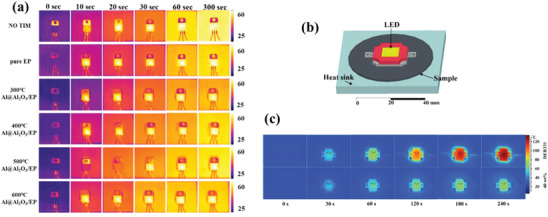
a) Evolution of the temperature of a MOSFET device with and without a TIM made of an Al@Al_2_O_3_‐epoxy composite versus time operating. Reproduced with permission.^[^ ^85^ ^]^ Copyright 2019, Elsevier. b) Structure diagram of the LED chip used.^[^ ^83^ ^]^ c) Evolution of the temperature of the LED chip versus time operating.^[^ ^83^ ^]^

The development of such polymer nanocomposites filled with core–shell fillers aims to cover several industrial fields. The most frequently cited applications are related to microelectronics and electronic packaging,^[^ ^31^, ^51^, ^57^, ^61^, ^70^, ^84^, ^86^, ^94^, ^95^, ^101^, ^102^ ^]^ and often linked with the miniaturization‐driven industry of electronics and microelectronics.^[^ ^50^, ^54^, ^58^, ^62^, ^67^, ^71^, ^72^, ^76^, ^77^, ^80^, ^81^, ^82^, ^91^, ^96^, ^99^, ^105^ ^]^ For instance, Thermal interface materials (TIMs) are often cited as targeted applications for the microelectronics industry.^[^ ^33^, ^35^, ^65^, ^78^, ^85^, ^97^, ^98^, ^106^ ^]^ Mao et al.^[^ ^85^ ^]^ tested their thermally dissipative composite materials on an actual MOSFET device as presented in **Figure** **8**a. Other publications target electrical and power systems^[^ ^52^, ^53^, ^55^, ^90^ ^]^ such as LED,^[^ ^60^, ^61^, ^83^ ^]^ energy storage systems,^[^ ^61^, ^82^, ^89^ ^]^ or communication equipment.^[^ ^34^, ^100^ ^]^ For instance, Xu et al.^[^ ^83^ ^]^ tested their core–shell Al@Al_2_O_3_@SiO_2_ filled epoxy composite on an actual LED device and observed the improvement of thermal dissipation through thermal images showing a better cooling of the LED device using their epoxy‐Al@Al_2_O_3_@SiO_2_ composite as a heat sink (Figure 8b,c).

## 4 Conclusions and Perspectives

Polymer composites are widely used, yet their performance remains too limited for certain heat dissipation applications, notably in electronics and electrochemical storage systems such as batteries. The intrinsic low thermal conductivity of polymers can be compensated for by dispersing fillers within the matrix. A large number of fillers have already been studied, but in the specific case where high thermal conductivity has to be combined with low electrical conductivity, very few solutions are proving relevant to date. A novel class of fillers has been developed by combining a thermally conductive core with a conformal shell that circumscribes the electrical conduction of the core while optimizing the interface between the filler and the matrix. The core–shell particles can be composed of a vast array of chemical natures, including ceramic, carbon, metal, or polymer. Preferentially, the core is made of metal, carbon, or ceramic, while the shell is made of ceramic or polymer. The chemical nature of the shell plays a pivotal role in thermal conduction by limiting interfacial thermal resistance. Its surface chemistry is as well of critical significance in ensuring optimal cohesion with the matrix. Furthermore, the various examples discussed in this review demonstrate that key parameters, such as shell thickness, nanoparticle form factor, or filler loading, have a significant impact on final performance.

In conclusion, this technology enables the fabrication of core–shell particle based composites with markedly superior properties, typically exhibiting thermal conductivities on the order of several W m^−1^ K^−1^ while maintaining their electrical insulation. This promising approach is highly scalable, given the wide range of materials that can be considered, and should pave the way for a multitude of new fillers for the fabrication of high‐performance heat dissipative polymer composites.

## Conflict of Interest

The authors declare no conflict of interest.

## Author Contributions

A.B. and A.C. conducted literature research and drafted the manuscript. T.P. and A.C. contributed to manuscript revision. J.P.S. drafted the final version of the manuscript. All authors discussed and approved the submission of the manuscript.
